# On the conservation of energy: Noether's theorem revisited

**DOI:** 10.1016/j.heliyon.2024.e27476

**Published:** 2024-03-07

**Authors:** Jean-Paul Chavas

**Affiliations:** Taylor Hall, University of Wisconsin, Madison, WI, 53706, USA

**Keywords:** Energy conservation, Noether's theorem, Nonconvexity, Hamiltonian

## Abstract

This paper studies the dynamics and conservation of energy. It evaluates the validity of Noether's theorem as a formal argument supporting the law of conservation of energy in physical systems. The analysis examines the role of nonconvexity in energy dynamics. The paper argues that nonconvexity can arise in the presence of catalytic effects or in situations of transitions between multiple regimes. With the introduction of nonconvexity, the analysis relies on a generalized Lagrangian and generalized Hamiltonian. The investigation applies under general conditions, allowing for multiple types of energy with dynamics driven by multiple state variables. Our key result is to show that the conservation of energy (Noether's theorem) holds under convexity but not under nonconvexity. This identifies situations where energy in isolated systems is not necessarily constant over time. By relaxing the law of conservation of energy, our analysis provides new insights into energy dynamics. It offers new directions for scientific inquiries, including improved understanding about the origin of life, the evolution of the early universe and the nature of space and time.

## Teaser

Significance: This paper studies the conservation of energy in the dynamics of physical systems, with a focus on the validity of Noether's theorem. The paper examines the role nonconvexity relying on a generalized Lagrangian and generalized Hamiltonian. The investigation applies under wide-ranging conditions, allowing for multiple types of energy with dynamics driven by multiple state variables. It shows that the law of conservation of energy (Noether's theorem) no longer holds under nonconvexity. Nonconvexity can arise in the presence of catalytic effects or in situations of transitions between multiple regimes. This identifies situations where energy in isolated systems is not necessarily constant over time. By relaxing the law of conservation of energy, our analysis provides new insights into energy dynamics. It offers new directions for scientific inquiries, including improved understanding about the origin of life, the evolution of the early universe and the nature of space and time.

## 1 Introduction

The conservation of energy has been seen as a fundamental law of nature. It states that energy in an isolated system can neither be created nor destroyed: it can only be transformed from one form to another. The law of energy conservation applies in general, going from Newtonian mechanics, to classical, relativistic and quantum mechanics. Arguments supporting the conservation of energy come from two sources. First, so far, there is no experiment showing strong empirical evidence against it. Second, the conservation of energy has been formally proven by Noether's theorem when the laws of physics do not change [1]. But there are many forms of energy, including mechanical, chemical, electrical, thermal and nuclear energy. This makes the assessment of energy transformations from one form to another somewhat difficult. The analysis of energy dynamics has typically focused on two types of energy: potential energy that is stored in objects in given position, and kinetic energy associated with motions. From Noether's theorem, a simple proof that energy is conserved can be obtained when the kinetic energy is a quadratic function of velocities and the potential energy does not depend on velocity ([2], p. 188). But could there be conditions under which energy conservation does not apply? Note that this question is not new: previous literature has addressed this issue by investigating how the arguments depend on how the conservation law is defined and the nature of energy dynamics [[3], [4], [5]]. This paper contributes to these inquiries with a focus on revisiting Noether's theorem under general conditions. Our analysis is general in the sense that it applies to multiple types of energy with dynamics involving multiple state variables. In this context, we show that Noether's theorem holds but only under convexity assumptions.

Our analysis of energy dynamics builds on three basic concepts that have been at the heart of physics and its historical development [[6], [7], [8], [9], [10]]:

C1: the time-invariance of the laws of physics,

C2: the principle of least action,

C3: the conservation of energy (Noether's theorem under C1).

The time invariance of the laws of physics (condition C1) applies to closed systems and assumes that dynamics of energy in an isolated system should be self-contained. The principle of least action (condition C2) assumes that the evolution of a physical system behaves in a way that is consistent with minimal effort [7,11,12]. Finally, the conservation of energy (C3) assumes that energy can neither be created nor destroyed; rather, it can only be transformed or transferred from one form to another [6,13]. Under conditions C1 and C2, our analysis examines the evolution of a closed physical system with a focus on the role of nonconvexity and its implications for condition C3.

In a minimization problem motivated under the least action principle (C2), it is typically assumed that the function being minimized is convex.^1^ Arguments supporting convexity are largely consistent with theoretical physics and its historical developments [2,[6], [7], [8], [9], [10]]. For example, kinetic energy is typically assumed to be a quadratic and convex function of velocities. Yet, there are at least two situations where convexity may not hold. The first situation arises in the presence of catalytic effects, where a small increase in a catalytic factor can generate a large positive effect on the objective function but only within a limited range, suggesting that catalytic factors would be associated with a non-convex objective function. The second situation involves dynamic systems displaying multiple regimes, where the convexity property holds within each regime but not in transitions between regimes. In this case, convexity would hold locally but not during transitions from one regime to another.

This paper examines the dynamics of energy when convexity may not apply. Under condition C1 and C2, the analysis is presented as a dynamic optimization problem and relies on optimal control methods. Under convexity, the approach makes use of Lagrangian and Hamiltonian as dual representations of the problem [14,15]. Theoretical physics has relied extensively on such dual formulations [2,[8], [9], [10]]. Our paper introduces nonconvexity in the analysis with an important difference: the dual formulation of the problem relies on a generalized Lagrangian and generalized Hamiltonian [16]. Under this generalized dual formulation, standard Lagrange multipliers no longer apply and are replaced by nonlinear functions capturing the effects of nonconvexity on dynamics. In this context, we explore how nonconvexity affects the conservation of energy (condition C3). When the laws of physics do not change over time, we show that the conservation of energy (as commonly stated in physics theory [1,6,13]) holds but only under convexity. Our analysis documents that the introduction of nonconvexity has significant effects on energy dynamics: in situations where nonconvexities arise, Noether's theorem does not hold and the law of conservation of energy no longer applies. Implications of our results are discussed.

## 2 Research methods

Consider an isolated physical system that evolves over time. As noted in the introduction, the analysis is applied to a closed system under condition C1 (time-invariance of the laws of physics) and condition C2 (principle of least action). In this context, the system dynamics is represented by a constrained minimization problem, with a focus on the evolution of energy over time. In turn, the optimal dynamics have a dual representation typically captured using a Lagrangian-Hamiltonian formulation. Such dual formulations are commonly found in classical mechanics [8,9,17], in special and general relativity [10] as well as in quantum mechanics [18].

At time t, consider a physical system involving n state variables $x_{t}\in X\subset R^{n}$ and k control variables $u_{t}\in U\subset R^{k}$, where $t\in T\equiv \left[t_{0},t_{1}\right]$, $t_{0}$ and $t_{1}$ denoting initial time and terminal time, respectively.^2^ For a given initial condition $x_{0}$, assume that $x_{t}$ evolves over time according to the differential equation:

(1) $$\frac{{dx}_{t}}{dt}\equiv {\dot{x}}_{t}=g\left(x_{t},u_{t}\right)$$

where $u_{t}\in U$ and $g:X\times U\to R^{n}$, t∈T. Under conditions C1 and C2, consider the minimization problem:

(2) $$V=\min_{x,u}\;\{\int_{t_{0}}^{t_{1}}\;f\left(x_{t},u_{t}\right)\;dt:{\dot{x}}_{t}=g\left(x_{t},u_{t}\right),u_{t}\in U,t\in T\}\text{,}$$

where $x=\{x_{t}:t\in T\}$ and $u=\{u_{t}\in U,t\in T\}$, and $f:R^{n}\times U\to R$. The function $f\left(x_{t},u_{t}\right)$ measures energy in the system at time t and V measures total energy over the period $\left[t_{0},t_{1}\right]$. The functions $f\left(x_{t},u_{t}\right)$ and $g\left(x_{t},u_{t}\right)$ are assumed to be independent of time. This is consistent with condition C1: the time-invariance of the laws of physics. Also, the minimization problem in (2) is consistent with the principle of least action C2 [7,11,12].

Throughout the paper, we assume that the functions f:X×U→R and $g:X\times U\to R^{n}$ are differentiable. Let $S\equiv \{\left(x,u\right):{\dot{x}}_{t}=g\left(x_{t},u_{t}\right),x_{t}\in X,u_{t}\in U,t\in T$} be the feasible set in (2). We also assume that S has a nonempty interior and that the set $\{\int_{t_{0}}^{t_{1}}\;f\left(x_{t},u_{t}\right)\;dt$: (x,u)∈S} has a lower bound, thus guaranteeing that problem (2) has a solution. Denote its solution by $\left(x^{*}.u^{*}\right)$, where $u^{*}=\{u_{t}^{*}:t\in T\}$ is a piecewise continuous optimal control path and $x^{*}=\{x_{t}^{*}:t\in T\}$ is the corresponding optimal state trajectory.

Note the generality of the approach. Given a system represented by the n-dimensional state vector $x_{t}$, the function $f\left(x_{t},u_{t}\right)$ in (2) provides a general measurement of the energy-momenta of the system at time t and the function $g\left(x_{t},u\right)$ in equation (1) provides a general representation of energy dynamics. This allows for multiple types of energy with dynamics involving multiple state variables. As noted in the introduction, we do not assume that the function f is convex, nor that the feasible set S is a convex set. As discussed below, allowing for nonconvexity has important implications in our analysis.

To obtain insights into energy dynamics, we seek to investigate a dual formulation to (2). Let $p_{t}\in P$ where P is the class of absolutely continuous functions $p_{t}:R^{n}\to R$ satisfying $p_{t}\left(0\right)=0,t\in T$, and with $p=\{p_{t}:t\in T\}$ being continuous over time. Consider the associated generalized Lagrangian:

(3) $$L\left(x,p,u\right)=\int_{t_{0}}^{t_{1}}\;\left[f\left(x_{t},u_{t}\right)+p_{t}\left(g\left(x_{t},u_{t}\right)\right)-p_{t}\left({\dot{x}}_{t}\right)\right]\;dt$$

Equation (3) a generalized Lagrangian; it would reduce to a standard Lagrangian in the special case where $p_{t}$ is taken to be linear for all t∈T, i.e. where $p_{t}\left(\beta \right)=\lambda_{t}\;\beta$, $\lambda_{t}\in R^{n}$ being standard Lagrange multipliers [15].

Equation (3) can be alternatively written as

(4a) $$L\left(x,p,u\right)=\int_{t_{0}}^{t_{1}}H\left(x_{t},p_{t},u_{t}\right)dt-\int_{t_{0}}^{t_{1}}\;p_{t}\left({\dot{x}}_{t}\right)\;dt$$

where $H\left(x_{t},p_{t},u_{t}\right)$ is defined as

(4b) $$H\left(x_{t},p_{t},u_{t}\right)=f\left(x_{t},u_{t}\right)+p_{t}\left(g\left(x_{t},u_{t}\right)\right)\text{.}$$

Equation (4b) a generalized Hamiltonian; it would reduce to a standard Hamiltonian in the special case where $p_{t}$ is taken to be linear for all t∈T, i.e. where $p_{t}\left(\beta \right)=\lambda_{t}\;\beta$, $\lambda_{t}\in R^{n}$ [15].

Consider a saddle-point $\left(x^{*},p^{*},u^{*}\right)$ of L(x,p,u) in (3), (4a), (4b) where $\left(x_{t}^{*},p_{t}^{*},u_{t}^{*}\right)\in X\times P\times U,t\in T$ satisfies

(5) $$L\left(x^{*},p,u^{*}\right)\leq L\left(x^{*},p^{*},u^{*}\right)\leq L\left(x,p^{*},u\right)$$

for all $\left(x_{t},p_{t},u_{t}\right)\in X\times P\times U,t\in T$. Following Gould [19], an important linkage between problems (2) and (5) is stated next. (All proofs are presented in Appendix A).

Lemma 1 Consider a saddle-point $\left(x^{*},p^{*},u^{*}\right)$ of L(x,p,u) in (5). Then $\left(x^{*},u^{*}\right)$ is a solution to problem (2) with $V=L\left(x^{*},p^{*},u^{*}\right)$.

Lemma 1 shows that a saddle-point of the generalized Lagrangian L(x,u,p) in (5) provides a dual representation to the primal problem (2). It states two important properties. First, it shows that finding a saddle-point of the generalized Lagrangian gives a sufficient condition to find a solution to problem (2). Second, the dual formulation introduces the functions $p_{t}\in P$. As discussed below, these dual functions provide additional information about the nature of energy dynamics in (2).

While the saddle-point problem in (5) is a sufficient condition to find a solution to problem (2), in general it is not a necessary condition. Indeed, there are scenarios where a saddle-point problem in (5) does not exist, in which case the strong duality result $V=L\left(x^{*},p^{*},u^{*}\right)$ would not hold and duality would break down. It means that some regularity conditions need to be imposed on problem (2) to guarantee strong duality. These conditions have been explored in previous literature [20,21]. Below, we assume that these conditions are satisfied.

The next result illustrates the usefulness of the dual formulation in (5).

Lemma 2 (Generalized minimum principle): If $\left(x^{*},p^{*},u^{*}\right)$ is a saddle-point of L(x,p,u) in (5), then for all t∈T, it satisfies^3^

(6a) $${\dot{x}}_{t}^{*}=g\left(x_{t}^{*},u_{t}^{*}\right)$$

(6b) $$\frac{\partial H}{\partial x_{t}}\left(x_{t}^{*},p_{t}^{*},u_{t}^{*}\right)+\frac{d}{\text{dt}}\left[\frac{\partial p_{t}^{*}}{\partial {\dot{x}}_{t}\;}\left({\dot{x}}_{t}^{*}\right)\right]=0$$

(6c) $$H\left(x_{t}^{*},p_{t}^{*},u_{t}^{*}\right)\leq H\left(x_{t}^{*},p_{t}^{*},u_{t}\right)\;\text{for}\;\text{all}\;u_{t}\in U\text{.}$$

Lemma 2 is a “generalized minimum principle”. Indeed, equation (6a), (6b), (6c) generalize some well-known results found in previous literature. Two special cases of (6a), (6b), (6c) are of particular interest. First, consider the case where equation (1) takes the form: $g\left(x_{t},u_{t}\right)=u_{t}$ where $u_{t}\in U\equiv R^{n}$. Then using (4b), an interior solution to the minimization problem in (6c) implies

$$\frac{\partial H}{\partial u_{t}}\left(x_{t}^{*},u_{t}^{*}\right)\equiv \frac{\partial f}{\partial u_{t}}\left(x_{t}^{*},u_{t}^{*}\right)+\frac{\partial p_{t}^{*}}{\partial {\dot{x}}_{t}\;}\left({\dot{x}}_{t}^{*}\right)=0,t\in T\text{.}$$

Substituting this result into equation (6b) and using (4b) yields the following result.

Corollary 1: Assume that $g\left(x_{t},u_{t}\right)=u_{t}$ and $U=R^{n}$ in (1). If $\left(x^{*},p^{*},u^{*}\right)$ is a saddle-point of L(x,p,u), then for all t∈T, equations (6a), (6b), (6c) reduce to

(7) $$\frac{\partial f}{\partial x_{t}}\left(x_{t}^{*},{\dot{x}}_{t}^{*}\right)-\frac{d}{\text{dt}}\left[\frac{\partial f}{\partial {\dot{x}}_{t}}\left(x_{t}^{*},{\dot{x}}_{t}^{*}\right)\right]=0,t\in T\text{.}$$

Equation (7) is the standard Euler-Lagrange equation of optimal control [15]. The Euler-Lagrange equation (7) has been a cornerstone in physics [6,7], including classical mechanics [2,8,9,17] and special and general relativity [10]. Corollary 1 states a nice result: the Euler-Lagrange equation is a special case of Lemma 2. But this result is obtained assuming that $g\left(x_{t},u_{t}\right)=u_{t}$, which is a rather restrictive condition for state dynamics. This indicates that equation (7) would not apply in situations where $g\left(x_{t},u_{t}\right)\neq u_{t}$. This identifies an important limitation of the Euler-Lagrange equation: equation (7) is not expected to hold in systems exhibiting more complex dynamics.

Second, consider the special case where $p_{t}\in P_{0}\subset P$ where $P_{0}$ is restricted to include only linear functions. In this case, $p_{t}\in P_{0}$ means that $p_{t}\left(\beta \right)$ can be written as $p_{t}\left(\beta \right)=\lambda_{t}\;\beta$. Then, the generalized Lagrangian in (3) becomes the standard Lagrangian $L_{0}\left(x,\lambda ,u\right)\equiv \int_{t_{0}}^{t_{1}}\;\left[f\left(x_{t},u_{t}\right)+\lambda_{t}\;g\left(x_{t},u_{t}\right))-\lambda_{t}\;{\dot{x}}_{t}\right]\;\text{dt}$, and the generalized Hamiltonian in (4b) becomes the standard Hamiltonian $H_{0}\left(x_{t},\lambda_{t},u_{t}\right)\equiv f\left(x_{t},u_{t}\right)+\lambda_{t}\;g\left(x_{t},u_{t}\right)$ where $\lambda_{t}\in R^{n}$ are Lagrange multipliers. In this context Lemma 2 gives the following result.

Corollary 2: Assume that $p\in P_{0}$ with $p_{t}\left(\beta \right)=\lambda_{t}\;\beta$ and $H_{0}\left(x_{t},\lambda_{t},u_{t}\right)\equiv f\left(x_{t},u_{t}\right)+\lambda_{t}\;g\left(x_{t},u_{t}\right),t\in T$. Then, for t∈T, equation (6a), (6b), (6c) become^4^:

(8a) $$\frac{\partial H_{0}}{\partial \lambda_{t}}\left(x_{t}^{*},\lambda_{t}^{*},u_{t}^{*}\right){=\dot{x}}_{t}^{*}$$

(8b) $$\frac{\partial H_{0}}{\partial x_{t}}\left(x_{t}^{*},\lambda_{t}^{*},u_{t}^{*}\right)=-{\dot{\lambda }}_{t}^{*}$$

(8c) $$H_{0}\left(x_{t}^{*},\lambda_{t}^{*},u_{t}^{*}\right)\leq H_{0}\left(x_{t}^{*},\lambda_{t}^{*},u_{t}\right)\;\text{for}\;\text{all}\;u_{t}\in U\text{.}$$

Equation (8a), (8b), (8c) are Pontryagin’s principle of optimal control [14,15,22]. This is another nice result: the Pontryagin principle of optimal control is a special case of Lemma 1-2. Comparing (6) and (8) shows that $\frac{\partial p_{t}^{*}}{\partial {\dot{x}}_{t}\;}\left({\dot{x}}_{t}^{*}\right)$ in (6b) plays the role of the Lagrange multipliers $\lambda_{t}^{*}$ in (8b). The interpretation of $\frac{\partial p_{t}^{*}}{\partial {\dot{x}}_{t}\;}\left({\dot{x}}_{t}^{*}\right)$ is discussed below.

Assuming that a saddle-point exists in (5) and using Lemma 1, equation (6a), (6b), (6c) in Lemma 2 are necessary conditions for $\left(x^{*},u^{*}\right)$ to be a solution to problem (2). Similar arguments apply to equation (7) in Corollary 1, or equation (8a), (8b), (8c) in Corollary 2. But in general, these equations are not sufficient. This raises the question: Under what conditions would these equations be both necessary and sufficient conditions to characterize a solution to problem (2)? This question has been addressed in the context of equation (8a), (8b), (8c) applied to a standard Lagrangian approach [15,23]. Mangasarian [23] and Luenberger [15] showed that equation (8a), (8b), (8c) can become sufficient conditions for $\left(x^{*},u^{*}\right)$ to be a solution to problem (2) under convexity, where convexity is defined as situations where f:X×U→R is a convex function and the feasible set S is convex. Indeed, under convexity, the separating hyperplane theorem applies establishing the existence of a separating hyperplane with slopes given by the Lagrange multipliers $\lambda^{*}$ [15, p. 219]. This argument indicates that, without convexity, equation (8a), (8b), (8c) would in general not be sufficient for $\left(x^{*},u^{*}\right)$ to be a solution to problem (2). As noted above, we do not assume that f is a convex function nor that the feasible set S is convex, i.e. we allow for nonconvexity. In this context, equation (8a), (8b), (8c) become less useful: they may no longer characterize the solution to problem (2). This is a situation where equation (6a), (6b), (6c) (developed under a generalized Lagrangian in (3)) have an important advantage compared to (8) (developed under a standard Lagrangian): from Lemma 1, the saddle-point problem (5) provide sufficient conditions for $\left(x^{*},u^{*}\right)$ to be a solution to problem (2) even under nonconvexity.

**Fig. 1:**
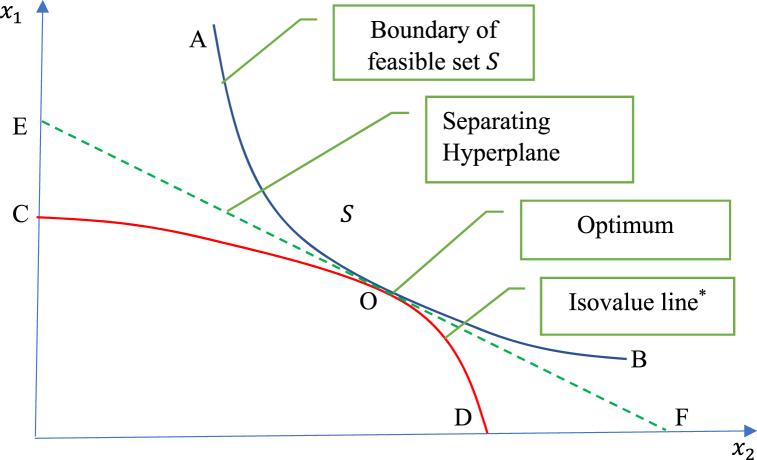
Optimality under convexity Note: The isovalue line is the set of points that gives the same value of the objective function as the one obtained at the optimum in the minimization problem (2).

**Fig. 2:**
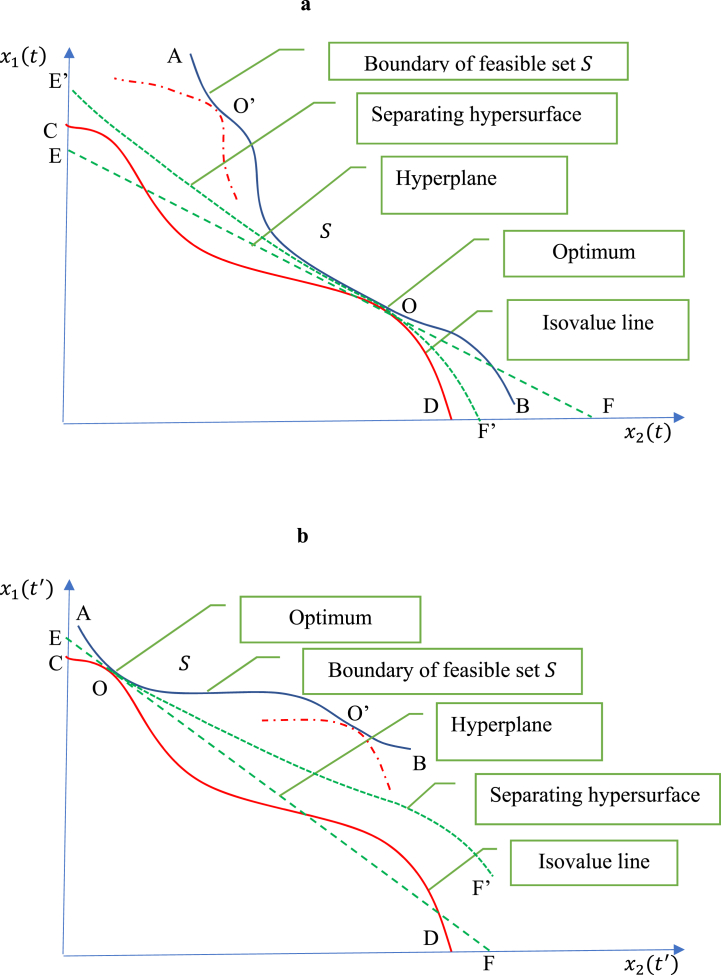
Optimality under nonconvexity, 2a: Evaluated at time t 2b: Evaluated at time $t^{\prime }$.

These arguments are illustrated in Fig. 1, Fig. 2. Fig. 1 presents a situation under convexity (where f is a convex function and the feasible set S is convex). In Fig. 1, the optimal solution is at point O. At this point, there is a separating hyperplane EOF that goes through point O while being tangent (at point O) to both the feasible set S (with AOB as a lower bound) and the isovalue line^5^ COD. In this case, convexity supports the existence of a separating hyperplane and of associated Lagrange multipliers; and convexity means that equation (8a), (8b), (8c) (developed under a standard Lagrangian approach) provide necessary and sufficient conditions to identify the optimal point O.

Fig. 2a-b present situations under nonconvexity where neither f is a convex function nor S is convex set. They identify two points O and O’. In Fig. 2a (evaluated at time t), the global solution to problem (2) is at point O and not at point O’. Point O’ is suboptimal (it provides only a “local solution” valid in the neighborhood of O’). Fig. 2a shows the presence of a hyperplane EOF that goes through point O while being tangent to both the feasible set S (with AOB as a lower bound) and the isovalue line COD. But under nonconvexity, the hyperplane EOF does not have the separation property: the hyperplane intersects the isovalue line COD when $x_{2}\left(t\right)$ is small; and it enters the feasible set S when $x_{2}\left(t\right)$ is large. This failure to have a separating hyperplane is due to non-convexity. As discussed below, this failure is important: it means that equation (8a), (8b), (8c) would be satisfied at both O and O’, reflecting that equation (8a), (8b), (8c) can fail to identify a solution to problem (2) (as point O’ is suboptimal). This provides the motivation to switch from a standard Lagrangian to the generalized Lagrangian in (3): switching from a linear $p_{t}\in P_{0}$ to a nonlinear $p_{t}\in P$ is equivalent to switching from a (non-separating) hyperplane EOF to a separating nonlinear hypersurface E’OF’ in Fig. 2a. Similar arguments apply to Fig. 2b (evaluated at time $t^{\prime }$).

In Fig. 2a-b, the hyperplane EOF and the separating hypersurface are both tangent to the lines AOB and COD, illustrating why equations (6a), (6b), (6c), (8a), (8b), (8c) would hold in the neighborhood of point O. Yet, allowing for nonlinear $p_{t}\in P$ in the generalized Lagrangian means that the slopes of $p_{t}$ are no longer constant. From Fig. 2a–b, a separating hyperplane may no longer exist under nonconvexity; but the separation property still applies to a nonlinear hypersurface. Under nonconvexity, the functions $p_{t}$ must be allowed to be nonlinear to guarantee the separation property. This shows why the standard Lagrangian approach is overly restrictive: without convexity, it is inappropriate to assume that the functions $p_{t}$ are linear (i.e., that the Lagrange multipliers are constant). This motivates switching to the generalized Lagrangian in (3) where the function $p_{t}$ are allowed to be nonlinear. In this case, the slopes of $p_{t}$ in (3) are not constant, i.e., its marginal values $\frac{\partial p_{t}\left(\beta \right)}{\partial \beta }$ can vary with the evaluation point β.

To stress the importance of switching to the generalized Lagrangian in (3), note that Lemma 1 guarantees to find a global solution (denoted by point O in Fig. 2a-b). Thus, Lemma 1 avoids the issue related to the existence of suboptimal points (such as O’ in Fig. 2a-b) where equation (7) or (8) would be satisfied. In addition, equation (6a), (6b), (6c) provide useful information about optimal dynamics under nonconvexity. This includes the dynamics of $p^{*}$ given in equation (6b) which involves the term $\frac{d}{\text{dt}}\left[\frac{\partial p_{t}^{*}}{\partial {\dot{x}}_{t}\;}\left({\dot{x}}_{t}^{*}\right)\right]$. When $p_{t}^{*}\left({\dot{x}}_{t}^{*}\right)$ is nonlinear (under nonconvexity), its slopes $\frac{\partial p_{t}^{*}\left(\beta \right)}{\partial \beta \;}$ would change with the evaluation point β. In this context, equation (6b) shows that the evolving slopes of $p_{t}^{*}\left({\dot{x}}_{t}^{*}\right)$ play an important role in energy dynamics.

To provide insights into the interpretation of $\frac{\partial p_{t}^{*}}{\partial {\dot{x}}_{t}\;}\left({\dot{x}}_{t}^{*}\right)$, consider the perturbation function:

(9) $$V\left(b\right)=\min_{x,u}\;\{\int_{t_{0}}^{t_{1}}\;f\left(x_{t},u_{t}\right)\;\text{dt}:{\dot{x}}_{t}=b_{t}+g\left(x_{t},u_{t}\right),u_{t}\in U,t\in T\}\text{,}$$

where $b=\{b_{t}:t\in T\}$ and $b_{t}$ denotes an exogenous change in $g\left(x_{t},u_{t}\right)$. From (2), noting that V=V(0), it follows that V(b) reflects the effects of a change in energy flows on the optimal total energy V. When the function V(b) is differentiable in b at point b=0, Chavas [16] showed that

(10) $$\frac{\partial V\left(b\right)}{\partial b_{t}}=\frac{\partial p_{t}^{*}}{\partial {\dot{x}}_{t}}{(\dot{x}}_{t}^{*})\text{.}$$

where $\frac{\partial p_{t}^{*}}{\partial {\dot{x}}_{t}}\left({\dot{x}}_{t}\right)$ is the derivative of $p_{t}^{*}\left({\dot{x}}_{t}\right)$ with respect to ${\dot{x}}_{t}$ at time t∈T. Equation (10) is a version of the envelope theorem, stating that $\frac{\partial p_{t}^{*}}{\partial {\dot{x}}_{t}}{(\dot{x}}_{t}^{*})$ is the marginal amount of energy generated by a small change in the states $x_{t}$ at time t. When $p^{*}\in P_{0}$ is linear, then $\frac{\partial p_{t}^{*}}{\partial {\dot{x}}_{t}}=\lambda_{t}^{*}$ and (10) reduces to the standard interpretation of Lagrange multipliers, with $\lambda_{t}^{*}$ measuring the marginal values of the constraints [15, p. 222]. Equation (10) generalizes this interpretation to situations where $p^{*}$ is nonlinear. With $\frac{\partial p_{t}^{*}}{\partial {\dot{x}}_{t}}{(\dot{x}}_{t}^{*})$ measuring the marginal rate of transformation of the states $x_{t}$ into energy and noting that $p_{t}^{*}\left({\dot{x}}_{t}^{*}\right)=\int_{\beta \in C\left({\dot{x}}_{t}^{*}\right)}\;\frac{\partial p_{t}^{*}\left(\beta \right)}{\partial \beta \;}\;d\beta$ where $C\left({\dot{x}}_{t}^{*}\right)$ is a smooth path of β from 0 to ${\dot{x}}_{t}^{*}$, it follows that ${p_{t}^{*}(\dot{x}}_{t}^{*})$ can be interpreted as a measure of the implicit energy generated by the states $x_{t}$ at time t∈T. Define

(11) $$I\equiv \int_{t_{0}}^{t_{1}}\;{p^{*}(\dot{x}}_{t}^{*})\;\text{dt.}$$

Using (10), the term I in (11) is the total implicit energy generated by the states $x_{t}$ during the period $\left[t_{0},t_{1}\right]$. Using equation (4a) evaluated at the optimum, we have

(12) $$V+I=\int_{t_{0}}^{t_{1}}\;{H(x}_{t}^{*},p_{t}^{*},u_{t}^{*})\;\text{dt}$$

The term (V+I) is the total generalized energy defined as actual total energy of the system $V=\int_{t_{0}}^{t_{1}}\;{f(x}_{t}^{*},u_{t}^{*})\;\text{dt}$ plus the total implicit energy I generated by the states over the period $\left[t_{0},t_{1}\right]$. It follows from (12) that the term $\int_{t_{0}}^{t_{1}}\;{H(x}_{t}^{*},p_{t}^{*},u_{t}^{*})\;\text{dt}$ can also be interpreted as the total generalized energy in the system. This gives the following useful interpretation of the Hamiltonian: at the optimum, the Hamiltonian ${H(x}_{t}^{*},p_{t}^{*},u_{t}^{*})$ provides a measure of the generalized energy associated with the system at time t. Thus, our analysis identifies the presence of three types of energy at time t: actual energy ${f(x}_{t}^{*},u_{t}^{*})$, implicit energy $p_{t}^{*}\left(g{(x}_{t}^{*},u_{t}^{*})\right)$, and generalized energy measured by the Hamiltonian ${H(x}_{t}^{*},p_{t}^{*},u_{t}^{*})\equiv {f(x}_{t}^{*},u_{t}^{*})+p_{t}^{*}\left(g{(x}_{t}^{*},u_{t}^{*})\right)$. This raises the question: what does the evolution of generalized energy tell us about energy conservation? This is the topic of the next section.

## 3 Results and discussion

As just discussed, we can study the conservation of energy by evaluating whether generalized energy (as measured by the Hamiltonian ${H(x}_{t}^{*},p_{t}^{*},u_{t}^{*})$) is constant over time. With a focus on the role of nonconvexity, our main result is stated next.

Proposition 1: Assume that $f\left(x_{t},u_{t}\right)$ and $g\left(x_{t},u_{t}\right)$ are independent of t and that $u_{t}^{*}\in \mathrm{int}\left(U\right),t\in T$. Evaluated at the optimum $\left(x_{t}^{*},p_{t}^{*},u_{t}^{*}\right)$,

a) If $p_{t}^{*}\left(\beta \right)=\lambda^{*}\;\beta$ for all t∈T, then $H_{0}\left(x_{t}^{*},\lambda_{t}^{*},u_{t}^{*}\right)$ is constant over time with

(13) $$\frac{dH_{0}\left(x_{t}^{*},\lambda_{t}^{*},u_{t}^{*}\right)}{\text{dt}}=0,t\in T\text{.}$$

b) If $p^{*}\left(\beta \right)\in P$ is a nonlinear function, t∈T, then $H\left(x_{t}^{*},p_{t}^{*},u_{t}^{*}\right)$ can vary over time t as it satisfies

(14) $$\frac{\text{dH}\left(x_{t}^{*},p_{t}^{*},u_{t}^{*}\right)}{\text{dt}}\left\{\begin{array}{c} >\\ =\\ < \end{array}\right\}0\;\text{as}\;\frac{dp_{t}^{*}}{\text{dt}}\left\{\begin{array}{c} >\\ =\\ < \end{array}\right\}\;\frac{d}{\text{dt}}\left[\frac{\partial p_{t}^{*}}{\partial {\dot{x}}_{t}\;}\left({\dot{x}}_{t}^{*}\right)\right]\frac{dx_{t}^{*}}{\text{dt}},t\in T\text{.}$$

Under condition C1, equation (13) is a well-known result: at the optimum in (2), the Hamiltonian $H\left(x_{t}^{*},\lambda_{t}^{*},u_{t}^{*}\right)$ is constant over time [24]. This is Noether's theorem, stating that the conservation of energy holds in an isolated system if the laws of physics do not change over time [1,13]. This result is obtained under a standard Lagrangian which applies under convexity (when the separating hyperplane theorem holds and $p_{t}^{*}$ is taken to be linear). But equation (14) gives a different result: it states that that the Hamiltonian $H\left(x_{t}^{*},p_{t}^{*},u_{t}^{*}\right)$ is not necessarily constant under a generalized Lagrangian approach where the function $p_{t}$ is allowed to be nonlinear. Equation (14) shows that $H\left(x_{t}^{*},p_{t}^{*},u_{t}^{*}\right)$ would $\left\{\begin{array}{c} \text{increase}\\ \text{decrease} \end{array}\right\}$ over time when $\frac{dp_{t}^{*}}{\text{dt}}\;\left\{\begin{array}{c} >\\ < \end{array}\right\}\;\frac{d}{\text{dt}}\left[\frac{\partial p_{t}^{*}}{\partial {\dot{x}}_{t}\;}\left({\dot{x}}_{t}^{*}\right)\right]\frac{dx_{t}^{*}}{\text{dt}}$, i.e. when the total shift in $p_{t}^{*}$ $\left\{\begin{array}{c} \text{exceeds}\\ \text{is}\;\text{less}\;\text{than} \end{array}\right\}$ its shift due to a slope change. The need for $p_{t}^{*}$ to be nonlinear arises under nonconvexity (as illustrated in Fig. 2). This is an important result: even when the laws of physics do not change, the conservation of energy no longer holds under nonconvexity. Note that, applied to generalized energy, these findings are obtained under wide-ranging conditions, allowing for multiple types of energy with dynamics driven by multiple state variables.

The results stated in Proposition 1 hold under conditions C1 (the time-invariance of the laws of physics) and condition C2 (the principle of least action). In this context, we obtain two important results: 1) while the standard Lagrangian approach is appropriate under convexity, the analysis needs to be extended to a generalized Lagrangian under nonconvexity; 2) under condition C1, while the conservation of energy applies under convexity, it does not necessarily hold under nonconvexity.

These results indicate that nonconvexity can play an important role in the dynamics of energy. Our analysis has several important implications. If the departure from convexity occurs only in specific neighborhoods, then it is possible that different dynamic patterns would develop in different neighborhoods. Importantly, this argument holds while keeping the laws of physics constant across time and space. A good example is given by apparent differences between quantum physics (holding in the large) versus relativistic physics (holding in the small) along with unsuccessful attempts to integrate them into a unified theory [25]. Addressing these issues raises several challenges. First, our analysis stresses the limitations of local dynamics: a good understanding of local dynamics can fail to provide an accurate representation of global dynamics. Second, we need to understand better how nonconvexity can generate apparent differences in dynamic patterns. Third, how could we use this information to generate hypotheses on how dynamics can vary across neighborhoods? Fourth, how could we obtain measurements that would help distinguish between local dynamics and global dynamics?

Nonconvexity also has implications for dynamics over time. This is illustrated in Fig. 2a-b. Fig. 2a and b shows the optimum (point O) at time t and $t^{\prime }$ respectively. The optimum in each Figure occurs in a different region of the state space, each region exhibiting its own local dynamics, with the two regions being separated by zones exhibiting nonconvexity. When $t^{\prime }>t$, it means that between t and $t^{\prime }$, the system dynamics must go through a transition phase. One possibility is that the system would stay in one region for some extended period (before time t), switch to another region between time t and $t^{\prime }$, and then stay there after time t’. In this case, due to nonconvexity, the dynamics would change significantly in the transition phase, even while the laws of physics remain unchanged. This is illustrated in Fig. 2a-b, showing that the dynamics are qualitatively different before, during and after the transition. This scenario seems to fit the story of cosmic inflation related to changing dynamics in the early universe, with an inflationary period believed to have occurred a fraction of a second after the Big Bang, period during which significant changes occurred in the relative role of gravity, electromagnetism, the strong and the weak nuclear forces [26]. These changing patterns are a reminder about the limitations of extrapolations beyond current observations. They also indicate a need to refine our understanding on how nonconvexity could contribute to shifting dynamics in the evolution of physical systems.

From Proposition 1, nonconvexity can play a significant role in the evolution of energy. Proposition 1a shows that, under convexity and assuming invariant laws of physics (condition C1), a physical system satisfying the least action principle (condition C2) would imply the conservation of energy (Noether's theorem). But Proposition 1b leaves open the possibility that energy could increase under some scenarios. Importantly, Proposition 1b applies only under a generalized Lagrangian-Hamiltonian (where the Lagrange multipliers are not constant). Since the need for a generalized Lagrangian-Hamiltonian arises only under nonconvexity, it follows that the prospects to see increases in energy (as measured by the generalized Hamiltonian) must be associated with nonconvexity. As noted in the introduction, this can happen in the presence of catalytic factors. First, by stimulating local energy transfers toward specific states, catalytic factors can generate local nonconvexity. Second, such transfers can possibly contribute to increased energy (at least in some neighborhoods). This scenario seems particularly relevant in evaluating the prospects for the origin of life (as life would be unlikely to develop in situations where energy is scarce). As stated in equation (14), the generalized Hamiltonian $H\left(x_{t}^{*},p_{t}^{*},u_{t}^{*}\right)$ can change over time when the marginal values of the states are nonlinear; and it would increase over time when $\frac{dp_{t}^{*}}{dt}>\;\frac{d}{dt}\left[\frac{\partial p_{t}^{*}}{\partial {\dot{x}}_{t}\;}\left({\dot{x}}_{t}^{*}\right)\right]\frac{dx_{t}^{*}}{dt}$, i.e. when the total shift in $p_{t}^{*}$ exceeds its shift due to a slope change. This can help identify situations where energy could increase over time. Such insights could prove useful in the search for life on exoplanets.

Finally, our analysis is based on the dynamic minimization problem in (2), where the objective function measures energy flows in a closed physical system. This is the primal problem where the n state variables $x_{t}$ reflect the evolution of energy-momenta in the system. The associated dual problem is given in equations (3), (4a), (4b), (5), involving the functions $p_{t}$ where $\frac{\partial p_{t}\left({\dot{x}}_{t}\right)}{\partial {\dot{x}}_{t}}$ measures the marginal rate of transformation of the states into energy at the optimum. As discussed in Cortes and Smolin [27], problem (2) may involve no space-time variables, no locality, no Planck length, and no uncertainty, while the dual variables could represent measures of space-time. In this case, energy-momenta would be treated as fundamental variables in the primal problem, while space-time would emerge from its dual formulation. As we argued, there is a need to consider $p_{t}$ to be nonlinear functions under nonconvexity. If the dual variables are emerging space-time variables, then space-time would exhibit nonlinear dynamics. Examining the linkages between nonconvexity and the dynamics of space-time is a good topic for future research.

## 4 Conclusion

Our analysis has shown that, while Noether's theorem holds under convexity, it does not hold under nonconvexity. As a result, the conservation of energy does not apply in general. We have argued that non-convex situations can arise in the presence of catalytic effects or in transitions between alternative regimes. By relaxing the law of conservation of energy, our analysis provides new insights into energy dynamics. It offers new directions for scientific inquiries, including improved understanding about the origin of life, the evolution of the early universe and the nature of space and time.

## Data availability statement

Data availability or data sharing is not applicable to this article as no data set was created or analyzed in this study.

## CRediT authorship contribution statement

**Jean-Paul Chavas:** Writing – review & editing, Writing – original draft, Project administration, Methodology, Investigation, Formal analysis, Conceptualization.

## Declaration of competing interest

The authors declare that they have no known competing financial interests or personal relationships that could have appeared to influence the work reported in this paper.

## Appendix A Proofs

Proof of Lemma 1: The first inequality in (5) implies that

(A1) $$\int_{t_{0}}^{t_{1}}\;\left[p_{t}\left(g\left(x_{t}^{*},u_{t}^{*}\right)\right)-p_{t}\left({\dot{x}}_{t}^{*}\right)\right]dt\leq \int_{t_{0}}^{t_{1}}\;\left[p_{t}^{*}\left(g\left(x_{t}^{*},u_{t}^{*}\right)\right)-p_{t}^{*}\left({\dot{x}}_{t}^{*}\right)\right]dt,\forall p_{t}\in P,t\in T\text{.}$$

Assume that $g\left(x_{t}^{*},u_{t}^{*}\right)-{\dot{x}}_{t}^{*}\neq 0$ for some t∈T. It follows that there exists a j∈[1,…,n] and an interval $\left[t_{a},t_{b}\right],t_{b}{>t}_{a}$, satisfying either $g_{j}\left(x_{t}^{*},u_{t}^{*}\right)-{\dot{x}}_{jt}^{*}>0$ or $g_{j}\left(x_{t}^{*},u_{t}^{*}\right)-{\dot{x}}_{jt}^{*}<0$ for all $t\in \left[t_{a},t_{b}\right]$. Let $t_{c}=0.5\;t_{a}+0.5\;t_{b}$. Consider $p_{t}\left(c\right)=0,\forall c$ and $p_{t}^{\prime }\left(c\right)=p_{t}\left(c\right)+k\;\left(t-t_{a}\right)\;1_{\left[t_{a},t_{c}\right]}\left(t\right)\;\left[g_{j}\left(x_{t}^{*},u_{t}^{*}\right)-{\dot{x}}_{jt}^{*}\right]+k\;\left(t_{b}-t\right)\;1_{\left(t_{c},t_{b}\right]}\left(t\right)\;\left[g_{j}\left(x_{t}^{*},u_{t}^{*}\right)-{\dot{x}}_{jt}^{*}\right]$, where $1_{A}\left(t\right)$ is an indicator function. Note that $p_{t}\in P$ and $p_{t}^{\prime }\in P$. Let $k\to \left\{\begin{array}{c} +\infty \\ -\infty \end{array}\right\}$ if $g_{j}\left(x_{t}^{*},u_{t}^{*}\right)-{\dot{x}}_{jt}^{*}\left\{\begin{array}{c} >0\\ <0 \end{array}\right\}$ for $t\in \left[t_{a},t_{b}\right]$. This would imply that $\int_{t_{0}}^{t_{1}}\;\left[p_{t}^{\prime }\left(g\left(x_{t}^{*},u_{t}^{*}\right)\right)-p_{t}^{\prime }\left({\dot{x}}_{t}^{*}\right)\right]\;dt\to \infty$, i.e. that $\int_{t_{0}}^{t_{1}}\;\left[p_{t}^{\prime }\left(g\left(x_{t}^{*},u_{t}^{*}\right)\right)-p_{t}^{\prime }\left({\dot{x}}_{t}^{*}\right)\right]\;dt$ does not have an upper bound. But this contradicts (A1). Thus, we conclude that

(A2) $$g\left(x_{t}^{*},u_{t}^{*}\right)-{\dot{x}}_{t}^{*}=0,\forall t\in T\text{.}$$

Using (A2), the second inequality in (5) states that

$$\int_{t_{0}}^{t_{1}}\;f\left(x_{t}^{*},u_{t}^{*}\right)dt\leq \int_{t_{0}}^{t_{1}}\;\left[f\left(x_{t},u_{t}\right)+p_{t}^{*}\left(g\left(x_{t},u_{t}\right)\right)-p_{t}^{*}\left({\dot{x}}_{t}\right)\right]\;dt,\forall \left(x_{t},u_{t}\right)\in X\times U,t\in T$$

(A3) $$=\int_{t_{0}}^{t_{1}}\;f\left(x_{t},u_{t}\right)\;dt,\forall \left(x,u\right)\in S\text{,}$$

which implies that $\left(x^{*},u^{*}\right)$ is a solution to problem (2). Using equations (A2), (A3), equations (2), (3) also imply the strong duality result: $V=L\left(x^{*},p^{*},u^{*}\right)$.

Proof of Lemma 2: Equation (6a) is equation (A2). Consider a feasible path $\left(x^{\prime },u^{\prime }\right)=\left(x^{*}+\delta_{x},u^{*}+\delta_{u}\right)\in S$, where $\left(\delta_{x},\delta_{u}\right)$ denotes small changes in (x,u). Using (4a), (4a) first-order Taylor series expansion in the neighborhood of $\left(x^{*},u^{*}\right)$, we have:

$$\delta L\left(x,p^{*},u\right)\equiv \int_{t_{0}}^{t_{1}}\;\left[H\left(x_{t}^{\prime },p_{t}^{*},u_{t}^{\prime }\right)-H\left(x_{t}^{*}{,p}_{t}^{*},u_{t}^{*}\right)-p_{t}^{*}\left({\dot{x}}_{t}^{\prime }\right)+p_{t}^{*}\left({\dot{x}}_{t}^{*}\right)\right]\;dt$$

$$\approx \int_{t_{0}}^{t_{1}}\;\left[\frac{\partial H}{\partial x_{t}}\left(x_{t}^{*\;},p_{t}^{*},u_{t}^{*}\right)\;\delta_{x_{t}}+H\left(x_{t}^{*},p_{t}^{*},u_{t}^{\prime }\right)-H\left(x_{t}^{*},p_{t}^{*},u_{t}^{*}\right)-\frac{{\partial p}_{t}^{*}}{\partial {\dot{x}}_{t}}\left({\dot{x}}_{t}^{*}\right)\;\delta_{{\dot{\dot{x}}}_{t}}\right]\;dt$$

(A4) $$=\int_{t_{0}}^{t_{1}}\;\left[\frac{\partial H}{\partial x_{t}}\left(x_{t}^{*\;},p_{t}^{*},u_{t}^{*}\right)\;\delta_{x_{t}}+H\left(x_{t}^{*},{p_{t}^{*},u}_{t}^{\prime }\right)-H\left(x_{t}^{*},{p_{t}^{*},u}_{t}^{*}\right)+\frac{d}{dt}\left[\frac{\partial p_{t}^{*}}{\partial {\dot{x}}_{t}\;}\left({\dot{x}}_{t}^{*}\right)\right]\delta_{x_{t}}\right]dt\;-\frac{{\partial p}_{t_{1}}^{*}}{\partial {\dot{x}}_{t_{1}}}\left({\dot{x}}_{t_{1}}^{*}\right)\;\delta_{x_{t_{1}}}+\frac{{\partial p}_{t_{0}}^{*}}{\partial {\dot{x}}_{t_{0}}}\left({\dot{x}}_{t_{0}}^{*}\right)\;\delta_{x_{t_{0}}}$$

using integration by parts. Using the second inequality in (5), we have $\delta L\left(x,p^{*},u\right)\geq 0$ for all feasible (x,u)∈S. From (A4), this implies

$$\left(\frac{\partial H_{t}}{\partial x_{t}}\left(x_{t}^{*\;},u_{t}^{*}\right)+\frac{d}{dt}\left[\frac{\partial p_{t}^{*}}{\partial {\dot{x}}_{t}\;}\left({\dot{x}}_{t}^{*}\right)\right]\right)\delta_{x_{t}}\geq 0$$

for all feasible $\delta_{x_{t}}$, which yields (6b).

Finally, note that the second inequality in (5) implies that $L\left(x^{*},p^{*},u^{*}\right)\leq L\left(x^{*},p^{*},u\right)$ for all u∈U. Then, equation (6c) follows from (4a).

Proof of Proposition 1: When $u_{t}^{*}\in \mathrm{int}\left(U\right)$, equation (6c) implies that $\frac{\partial H}{\partial u_{t}\;}\left(x_{t}^{*},p_{t}^{*},u_{t}^{*}\right)=0$, and equation (8c) implies that $\frac{\partial H_{0}}{\partial u_{t}\;}\left(x_{t}^{*},\lambda_{t}^{*},u_{t}^{*}\right)=0,t\in T$.

Case a) If $p_{t}\in P$ is linear for all t∈T, we have $p_{t}\left(\beta \right)=\lambda_{t}\;\beta$ and $H_{0}\left(x_{t},{\lambda_{t},u}_{t}\right)=f\left(x_{t},u_{t}\right)+\lambda_{t}\;g_{t}\left(x_{t},u_{t}\right)$ where $\lambda_{t}$ are standard Lagrange multipliers, t∈T. Then, evaluated at t∈T and $\left(x^{*},\lambda^{*},u^{*}\right)$,

$$\frac{dH_{0}\left(x_{t}^{*},\lambda_{t}^{*},u_{t}^{*}\right)}{dt}=\frac{\partial H_{0}}{\partial x_{t}}\left(x_{t}^{*},\lambda_{t}^{*},u_{t}^{*}\right)\frac{dx_{t}}{dt}+\frac{\partial H_{0}}{\partial \lambda_{t}}\left(x_{t}^{*},\lambda_{t}^{*},u_{t}^{*}\right)\frac{d\lambda_{t}}{dt}+\frac{\partial H_{0}}{\partial u_{t}}{(x}_{t}^{*},\lambda_{t}^{*},u_{t}^{*})\frac{du_{t}}{dt}$$

$=-{\dot{\lambda }}_{t}^{*}\;{\dot{x}}_{t}^{*}+$${\dot{x}}_{t}^{*}$${\dot{\lambda }}_{t}^{*}$ using equations (8a), (8b), (8c)

=0,which is equation (13).

Case b) If $p_{t}\in P$ is nonlinear, t∈T, then $H\left(x_{t},{p_{t},u}_{t}\right)=f\left(x_{t},u_{t}\right)+p_{t}\left(g_{t}\left(x_{t},u_{t}\right)\right)$ and

$$\frac{dH\left(x_{t}^{*},p_{t}^{*},u_{t}^{*}\right)}{dt}=\frac{\partial H}{\partial x_{t}}\left(x_{t}^{*},p_{t}^{*},u_{t}^{*}\right)\frac{dx_{t}}{dt}+\frac{\partial H}{\partial p_{t}}\left(x_{t}^{*},p_{t}^{*},u_{t}^{*}\right)\frac{dp_{t}}{dt}+\frac{\partial H}{\partial u_{t}}{(x}_{t}^{*},p_{t}^{*},u_{t}^{*})\frac{du_{t}}{dt}$$

$=-\frac{d}{dt}\left[\frac{\partial p_{t}^{*}}{\partial {\dot{x}}_{t}\;}\left({\dot{x}}_{t}^{*}\right)\right]\frac{dx_{t}^{*}}{dt}+\frac{dp_{t}^{*}}{dt}$ using equations (6a)-(6c)

$$\left\{\begin{array}{c} >\\ =\\ < \end{array}\right\}0\;\text{if}\;\frac{dp_{t}^{*}}{dt}\left\{\begin{array}{c} >\\ =\\ < \end{array}\right\}\frac{d}{dt}\left[\frac{\partial p_{t}^{*}}{\partial {\dot{x}}_{t}\;}\left({\dot{x}}_{t}^{*}\right)\right]\frac{dx_{t}^{*}}{dt}\text{,}$$

which is equation (14).

## Footnotes

1 Letting X be a convex subset of $R^{n}$, a differentiable function h:X→R is convex if and only if it satisfies $h\left(y\right)\geq h\left(x\right)+\frac{\partial h\left(x\right)}{\partial x}\;\left(y-x\right)$ for all x,y∈X; and for b∈R, h being a convex function implies that the set {x:h(x)≤b,x∈X} is a convex set.

2 More generally, $x_{t}\in X$ could be generalized coordinates defined in a n-dimensional manifold X.

3 In the case where the terminal time $t_{1}$ is free, using (A4) in Appendix A, the following equation would be added to (6a)-(6c): $\frac{{\partial p}_{t_{1}}^{*}}{\partial {{\dot{x}}_{t}}_{1}}=0$.

4 From footnote 3, if the terminal time $t_{1}$ is free, the following equation would be added to (8a)-(8c): $\lambda_{t_{1}}^{*}=0$

5 The isovalue line is the set of points that gives the same value of the objective function than the one obtained at the optimum in the minimization problem (2).
